# Prognostic value of ASXL1 mutations in patients with primary myelofibrosis and its relationship with clinical features: a meta-analysis

**DOI:** 10.1007/s00277-020-04387-7

**Published:** 2021-01-02

**Authors:** Ziqing Wang, Weiyi Liu, Mingjing Wang, Yujin Li, Xueying Wang, Erpeng Yang, Jing Ming, Richeng Quan, Xiaomei Hu

**Affiliations:** 1 Graduate School, Beijing University of Chinese Medicine, Beijing, 100029 China; 2 Department of Hematology, Xiyuan Hospital, China Academy of Chinese Medical Sciences, Beijing, 100091 China; 3 Graduate School, China Academy of Chinese Medical Sciences, Beijing, 100700 China

**Keywords:** ASXL1 mutations, Primary myelofibrosis, Prognostic value, Clinical features, Meta-analysis

## Abstract

Additional sex combs like 1 (ASXL1) mutations are one of the most common molecular biological abnormalities in patients with primary myelofibrosis (PMF), and the effect of these mutations on prognosis remains controversial. Hence, we conducted a meta-analysis to assess the prognostic value and clinical characteristics of ASXL1 mutations in PMF patients. Eligible studies were systematically searched from PubMed, Embase, and the Cochrane Library. We extracted the hazard ratios (HRs) and their 95% confidence intervals (CIs) of overall survival (OS) and leukemia-free survival (LFS), the number of patients transformed to acute leukemia, and clinical characteristics to carry out a meta-analysis by fixed effect model or random effect model according to the heterogeneity between studies. A total of 4501 PMF patients from 16 cohorts of 14 studies were included in this meta-analysis. The results revealed that ASXL1 mutations might predict a shorter OS (HR = 2.30, 95% CI: 1.79–2.94, *P* < 0.00001) and a higher probability of transformation to acute leukemia (LFS: HR = 1.77, 95% CI: 1.30–2.42, *P* = 0.0003; the rate of acute leukemia transformation: OR = 2.06, 95% CI: 1.50–2.83, *P* < 0.00001). Furthermore, ASXL1 mutations were correlated with patients older than 65 years old, male, a lower level of platelet counts, and a higher risk of the international prognostic score system. These findings indicate that ASXL1 mutations have a significant adverse impact on the prognosis of PMF patients and may contribute to risk stratification and prognostic assessment for PMF patients.

## Introduction

Primary myelofibrosis (PMF) is a kind of breakpoint cluster region protein (BCR)-Abelson tyrosine-protein kinase (ABL)–negative myeloproliferative neoplasm (MPN) resulted from the clonal proliferation of abnormal hematopoietic stem cells. It is mainly characterized by bone marrow fibrodysplasia, severe anemia, splenomegaly, constitutional symptoms (fatigue, night sweats, fever, cachexia), extramedullary hematopoiesis, progression to leukemia, and short survival [1]. In the US Surveillance, Epidemiology, and End Results (SEER) database, the incidence of PMF is 0.31/100,000, and the median age of onset is about 70 years old [2], whose survival is much lower than that of polycythemia vera and essential thrombocythemia, and it is the most aggressive among the Philadelphia chromosome-negative (Ph-) MPNs [3]. At present, the commonly used prognostic score systems for PMF in the clinic include the International Prognostic Scoring System (IPSS), Dynamic International Prognostic Scoring System (DIPSS), and DIPSS-Plus [4–6], which all focus on the clinical characteristics and karyotypes of patients, and have been widely used in clinical prognostic assessment and treatment guidance. In recent years, more and more studies have begun to pay attention to the role of gene mutations in the pathogenic mechanism of hematological diseases, and its influence on the progression and survival of PMF has gradually emerged; in the meantime, the traditional prognostic stratification method needs to be improved urgently [7–10]. With the widespread application of next-generation sequencing technology in hematological malignancies, studies found that besides MPN driver gene mutations (JAK2, CALR, MPL), there were often other gene mutations in PMF patients, including histone modification genes (ASXL1 and EZH2), RNA splicing factor genes (SRSF2, U2AF1, and SF3B1), DNA methylation (DNMT3A, TET2, and IDH1/2), signal transduction genes (CBL and NRAS), and DNA repair genes (TP53), which may coexist with driver gene mutations or in patients without driver gene mutations, and some patients may carry two or more non-driver gene mutations at the same time, some of which may affect the evolution and prognosis of PMF [11–13].

Additional sex combs like 1 (ASXL1) is one of the most common somatic mutant genes. It has been reported that about 13.04 to 37.8% of PMF patients have ASXL1 mutation [14–17]. The addition sex combs (ASX) gene was initially discovered from the genetic screening of Drosophila [18]. The human genome contains three Asx-like genes, ASXL1, ASXL2, and ASXL3. The ASXL1 gene is located on chromosome 20q11, including 13 exons and 12 introns, coding nucleoprotein, whose main structure contains the amino terminal ASX homology domain (ASXH) and the carboxy terminal plant homeodomain (PHD), and it is an enhancer of epigenetic regulatory proteins polycomb family and trithorax family, playing an important role in maintaining the stability of gene expression [18]. ASXL1 mutations include frameshift mutations, nonsense mutations, and missense mutations, of which frameshift are the most frequently happened mutations [19]. Studies have displayed that ASXL1 mutations have been found in patients with a variety of hematological malignancies, including MPN, myelodysplastic syndrome (MDS), acute myeloid leukemia (AML), and chronic myelomonocytic leukemia (CMML) [20–24].

In recent years, a large number of clinical studies on the impact of ASXL1 mutations in PMF have been reported, some of which have revealed that ASXL1 mutations may be related to the prognosis of PMF and leukemia transformation, but its exact role remains controversial. Many studies have suggested that ASXL1 mutations are predictive of poor OS [25–27] and a high risk of acute leukemia transformation for PMF patients [28–30], which are adverse factors for the prognosis of PMF patients. Nevertheless, they have also been shown to have no pronounced effect on OS and acute leukemia transformation [31–33]. Therefore, we performed a meta-analysis on data from relevant published studies to further explore the comprehensive prognostic value of ASXL1 mutations in PMF patients.

## Materials and methods

### Search strategy

Relevant literatures were systematically searched from PubMed, Embase, and the Cochrane Library from the establishment of the library to October 15, 2020, by using the following search terms: (“Primary Myelofibrosis” OR “Myelofibroses, Primary” OR “Myelofibrosis, Primary” OR “Primary Myelofibroses” OR “Bone Marrow Fibrosis” OR “Bone Marrow Fibroses” OR “Fibroses, Bone Marrow” OR “Fibrosis, Bone Marrow” OR “Myelofibrosis” OR “Myelofibroses” OR “Idiopathic Myelofibrosis” OR “Myeloid Metaplasia” OR “Metaplasia, Myeloid” OR “Metaplasias, Myeloid” OR “Myeloid Metaplasias” OR “Myelosclerosis” OR “Myeloscleroses” OR “Myelosis, Nonleukemic” OR “Myeloses, Nonleukemic” OR “Nonleukemic Myeloses” OR “Nonleukemic Myelosis” OR “Chronic Idiopathic Myelofibrosis” OR “Agnogenic Myeloid Metaplasia” OR “Agnogenic Myeloid Metaplasias” OR “Metaplasia, Agnogenic Myeloid” OR “Metaplasias, Agnogenic Myeloid” OR “Myeloid Metaplasia, Agnogenic” OR “Myeloid Metaplasias, Agnogenic” OR “Myelofibrosis With Myeloid Metaplasia” AND (“ASXL1” OR “additional sex combs like 1”). The references of the included studies were screened for more information. The review protocol has been registered in the PROSPERO International Prospective Register of Systematic Reviews (registration number: CRD42020214861).

### Selection criteria

Studies were included in the meta-analysis if they met the following criteria: (1) original articles were prospective or retrospective cohort studies and clinical trials; (2) assessed the prognostic effect of ASXL1 mutations in PMF patients; (3) provided data on overall survival (OS), leukemia-free survival (LFS) or the number of cases transformed to acute leukemia, from which we could get the hazard ratios (HRs) and their 95% confidence intervals (CIs) or sufficient data to estimate; (4) papers published in English. Review articles, case reports, meeting abstracts, animal studies, comments, and re-analyses were all excluded. However, we included one letter and two correspondences which fulfilled all of the inclusion criteria. The literature search and screening were conducted independently by 2 investigators (Ziqing Wang and Mingjing Wang). In case of disagreement, the opinions of the third investigator (Yujin Li) will be sought, and the best plan will be determined after discussion.

### Data extraction

Two reviewers (Ziqing Wang and Mingjing Wang) extracted the relevant data from the included articles independently. The following data were extracted from the articles: the first authors’ name, year of publication, study region, number of patients, median age and gender distribution of patients, diagnostic criteria of PMF, number and proportion of ASXL1 mutant patients, white blood cells (WBC), hemoglobin (HGB), platelet (PLT) count, IPSS and DIPSS-plus classification, number of patients with unfavorable karyotypes and transformed to acute leukemia, and HRs and 95% CIs for the OS and LFS based on ASXL1 mutation status. If the article reports multiple HRs of univariate analysis and multivariate analysis, the result of multivariate analysis is given priority because they may be more accurate.

### Quality assessment

Two reviewers (Ziqing Wang and Mingjing Wang) assessed the methodological quality of each included study with the Newcastle-Ottawa quality assessment scale (NOS) [34] for cohort studies independently. NOS is divided into three dimensions: selection, comparability, and outcome, which could be awarded a maximum of 4, 2, and 3 stars respectively, corresponding to the 9 items. Those with a final score of six stars or more were considered high-quality articles.

### Statistical analysis

All analyses were performed using STATA 14.0 (Stata Corporation, College Station, TX, USA) and Review Manager 5.3 (the Cochrane Collaboration, Oxford, UK). A bilateral *P* value of < 0.05 was defined as statistically significant. For the OS and LFS, HRs and corresponding 95% CIs were used to assess the prognostic effect of ASXL1 mutations in PMF patients. Besides, compared to ASXL1 wild-type PMF patients, HR > 1 suggested a poorer prognosis in ASXL1 mutation patients. The data of dichotomous variables were represented by odds ratio (OR) and its 95% CI, and the data of continuous variables were described by mean difference (MD) and 95% CI. The *Q* test (*P* < 0.10 was considered significant heterogeneity) and *I*^2^ statistic (*I*^2^ = 0–25%: no heterogeneity; *I*^2^ = 25–50%: moderate heterogeneity; *I*^2^ = 50–75%: large heterogeneity; *I*^2^ = 75–100%: extreme heterogeneity) were used to test the heterogeneity of the included studies. When *P* > 0.1 or *I*^2^ < 50%, the heterogeneity of the study was considered to be not statistically significant, and a fixed-effects model was used for analysis; otherwise, the heterogeneity of the study was considered to be statistically significant. Further sensitivity analysis or subgroup analysis was performed to analyze the source of heterogeneity; if heterogeneity still exists, a random-effects model would be used for meta-analysis. Sensitivity analysis was used to investigate the influence of each study on the overall HR, and Begg’s and Egger’s tests were conducted to detect the potential publication biases of included studies.

## Results

### Study selection

**Fig. 1:**
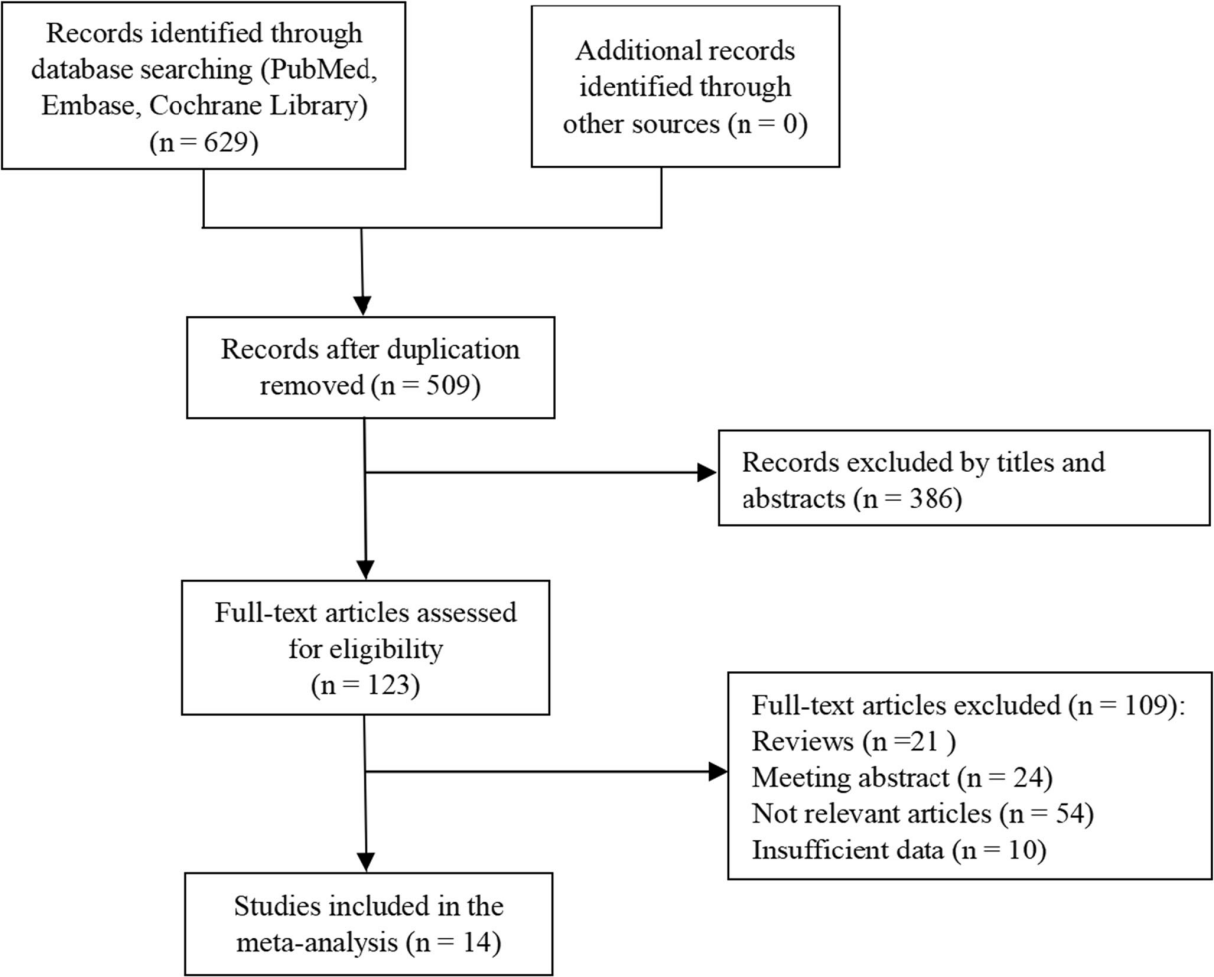
Flow diagram of study selection

As shown in Fig. 1, a total of 629 articles related to the above search terms were retrieved. After removing duplicate articles, according to the inclusion and exclusion criteria, 123 studies were obtained by reviewing the titles and abstracts. After reading the full text, 109 articles were excluded, including reviews, meeting abstracts, research irrelevant to the subject, and articles with insufficient data. Ultimately, 14 studies that met the criteria were finally included in this meta-analysis [8, 9, 24–33, 35, 36] (Fig. 1).

### Characteristics of included studies

Fourteen studies containing 4501 PMF patients were included in the meta-analysis, in which ASXL1 mutations were identified in 1393 PMF patients. The total mutation frequency was 30.95%. The sample size ranged from 45 to 661, and the frequency of ASXL1 mutations varied between 19.45 and 47.67%. Fourteen studied included 16 research cohorts (an article covered training and validation set and another article contained two groups of patients in pre-PMF and overt-PMF). One study originated from multiple research centers, five from the USA, eight from Europe, and two from Asia. Patients in 15 eligible studies were classified by the WHO criteria, and data from Tefferi 2016 were not available. The characteristics are listed in Table 1.

**Table 1:** Summary of the data extracted from the 16 studies included

First author	Year	Region	Patients(*n*)	ASXL1 mutations, *n* (%)	Median age year (range)	Sex (male/female)	Diagnostic criteria
Courtier [31]	2020	France	86	41 (48)	NA	NA	WHO
Gill [32]	2019	China	70	25 (36)	60 (26–89)	47/23	WHO
Guglielmelli [24]	2018	Italy	490	113 (23)	55.4 (14.0–70.0)	284/206	WHO
Guglielmelli [8]	2017	Italy ^#^	278	50 (18)	56.6 (18–90.3)	156/122	WHO
	2017	Italy ^*^	383	129 (34)	63.6 (14–89.8)	249/134	WHO
Yonal-Hindilerden [33]	2015	Turkey	77	19 (25)	60.8	34/43	WHO
Rotunno [28]	2019	Italy	333	119 (36)	NA	218/115	WHO
Song [29]	2017	USA	45	18 (40)	NA	NA	WHO
Tefferi [25]	2014	USA ^#^	277	85 (31)	64 (32–87)	177/100	WHO
	2014	Italy*	293	57 (19)	61.8 (14–90)	180/113	WHO
Tefferi [26]	2016	USA	182	65 (36)	63(22–87)	118/64	NA
Tefferi^##^ [27]	2018	USA	145	34 (23)	56 (22–87)	84/61	WHO
Tefferi** [9]	2018	USA/Italy	641	242 (38)	63 (−)	410/231	WHO
Vallapureddy [30]	2019	USA	596	246 (41)	NA	NA	WHO
Vannucchi [35]	2013	Europe	483	105 (22)	61 (14–90)	296/187	WHO
Wang [36]	2020	China	122	45 (37)	61 (21–88)	68/54	WHO

^#^One cohort of the study; *Another cohort of the study; ^##^*British Journal of Haematology*; **Leukemia; *NA*, not available; *WHO*, World Health Organization

### Quality assessment

The NOS was used to assess the quality of the included studies. There are 10 studies that scored 8 stars, 3 studies with 7 stars, and 1 study with 6 stars, indicating that the included studies were all high-quality articles. The detailed scores are shown in Table 2.

**Table 2:** Quality assessment of individual study

Study	Selection				Comparability	Outcome			Score
	Representativeness of exposed cohort	Selection of non-exposed cohort	Ascertainment of exposure	Outcome not present at start		Assessment of outcome	Follow-up length	Follow-up adequacy	
Courtier 2020 [31]	*	*	*	*	*	*	*	*	8
Gill 2019 [32]	*	*	*	*	*	*	*	*	8
Guglielmelli 2017 [8]	*	*	*	*	*	*	*	*	8
Guglielmelli 2018 [24]	*	*	*	*	*	*	*	*	8
Yonal-Hindilerden 2015 [33]	*	*	*	*	*	*	*	*	8
Rotunno 2019 [28]	*	*	*	*	–	*	*	*	7
Song 2017 [29]	*	*	*	*	*	*	–	*	7
Tefferi 2014 [25]	*	*	*	*	*	*	–	*	7
Tefferi 2016 [26]	*	*	*	*	*	*	*	*	8
Tefferi 2018^##^ [27]	*	*	*	*	*	*	*	*	8
Tefferi 2018** [9]	*	*	*	*	*	*	*	*	8
Vallapureddy 2019 [30]	*	*	*	*	*	*	*	*	8
Vannucchi 2013 [35]	*	*	*	*	*	*	*	*	8
Wang 2020 [36]	*	*	*	*	–	*	–	*	6

^##^*British Journal of Haematology*; **Leukemia

### Prognostic impact of ASXL1 mutations in patients with PMF

**Fig. 2:**
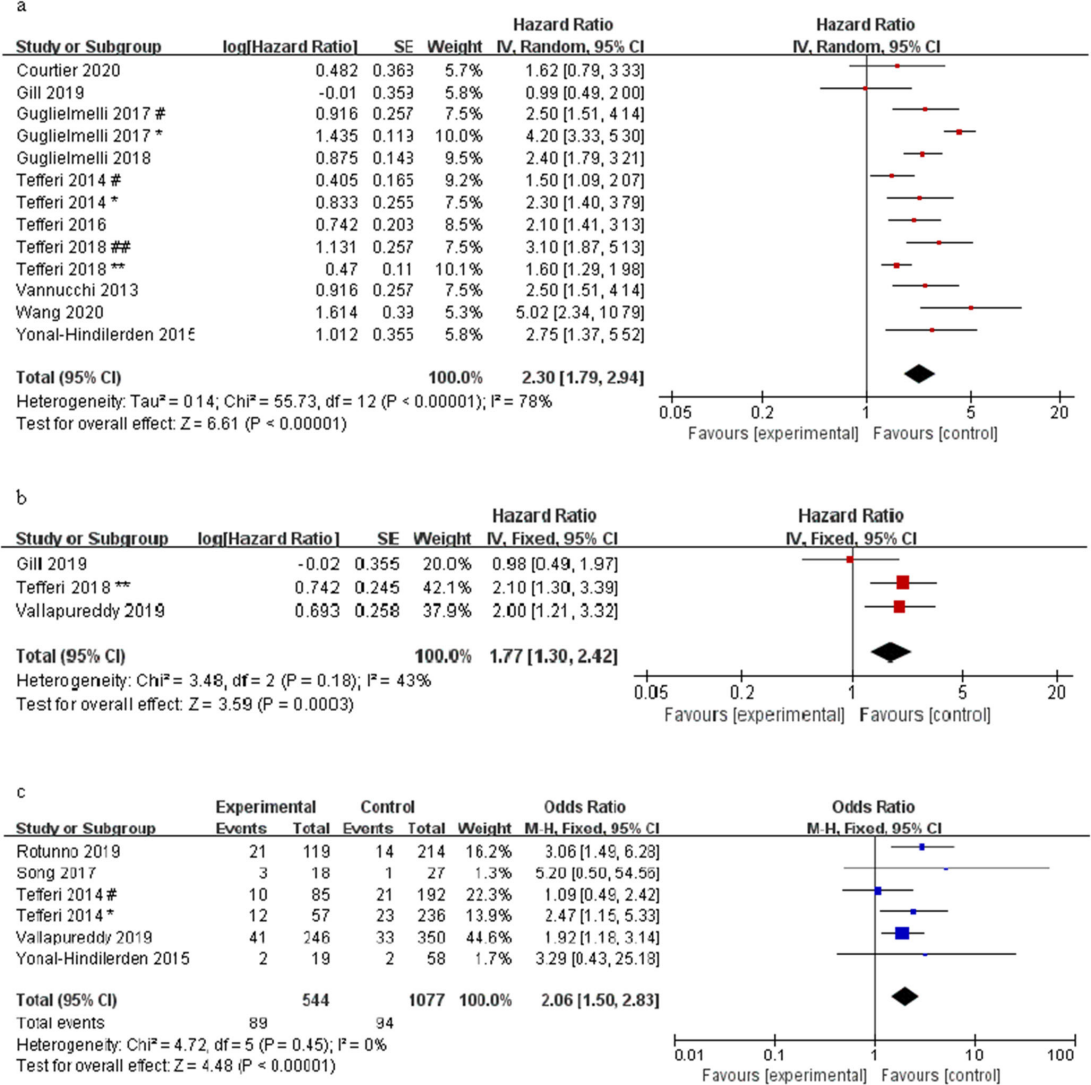
Forest plot of the pooled HRs and 95% CIs assessing the prognostic value of ASXL1 mutations in patients with PMF. **a** For OS by a random-effects model. **b** For LFS by a fixed-effects model. **c** For the rate of acute leukocyte transformation by a fixed-effects model. Number sign, one cohort of the study; single asterisk, another cohort of the study; double number sign, *British Journal of Haematology*; double asterisk, *Leukemia*

There were 13 studies involving OS, including 3527 patients. As shown in Fig. 2a, the overall HR for the OS was 2.30 (95% CI: 1.79–2.94, *I*^2^ = 78%, *P* < 0.00001) in PMF patients with ASXL1 mutations compared to those with ASXL1 wild-type. It indicated that ASXL1 mutations could predict a poorer OS in PMF patients.

HR was pooled for LFS extracted from 3 eligible studies. The overall HR for LFS was 1.77 (95% CI: 1.30–2.42, *I*^2^ = 43%, *P* = 0.0003) in ASXL1 mutation PMF patients compared with ASXL1 wild-type (Fig. 2b). Six studies reported the number of patients transformed to acute leukemia in ASXL1 mutations and wild-type PMF patients. Combining the effect size, the result of OR was 2.06 (95% CI: 1.50–2.83, *I*^2^ = 0%, *P* < 0.00001). The above two results of ASXL1 mutations might predict a higher probability of transformation to leukemia (Fig. 2c).

### Subgroup analysis and heterogeneity exploration

**Fig. 3:**
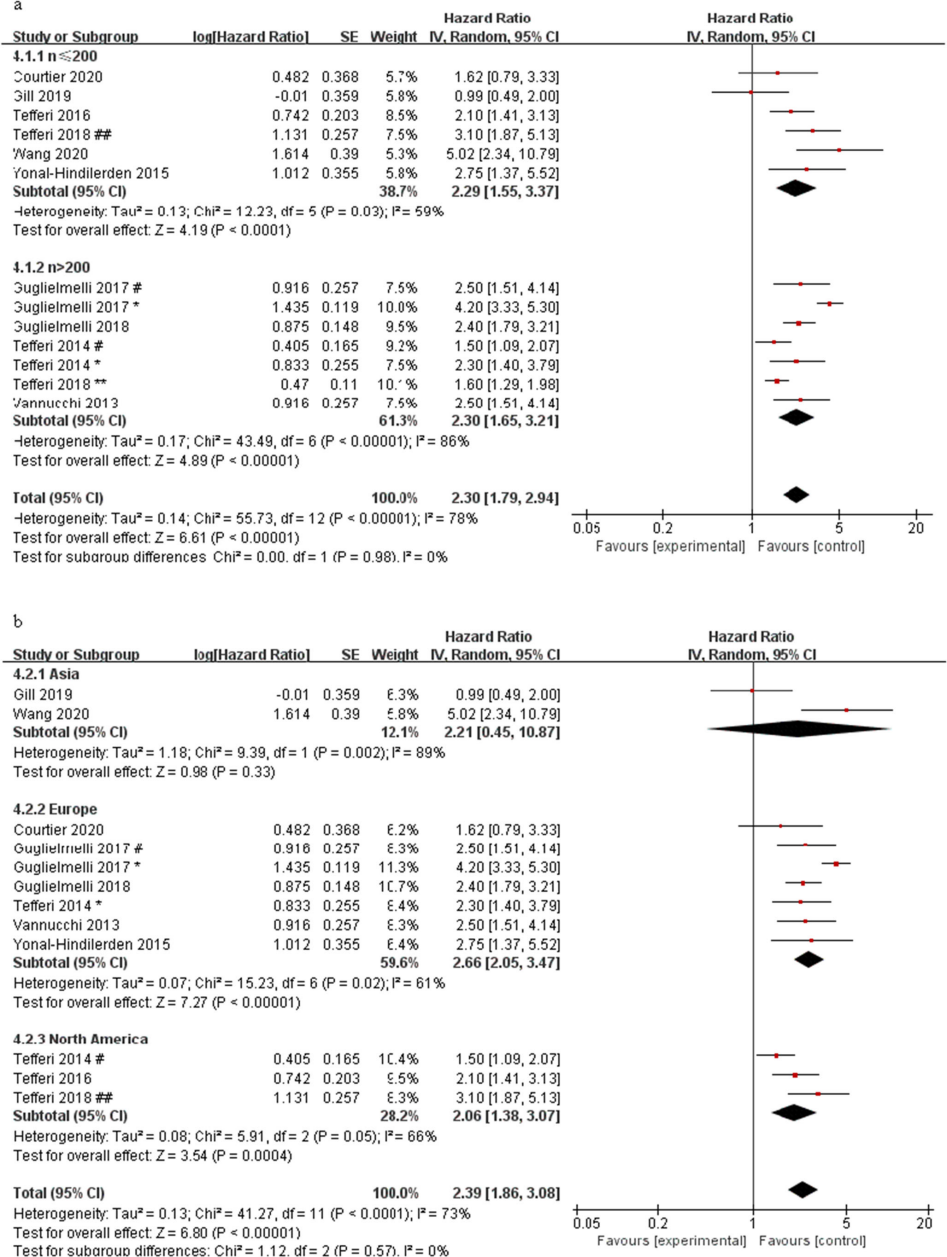

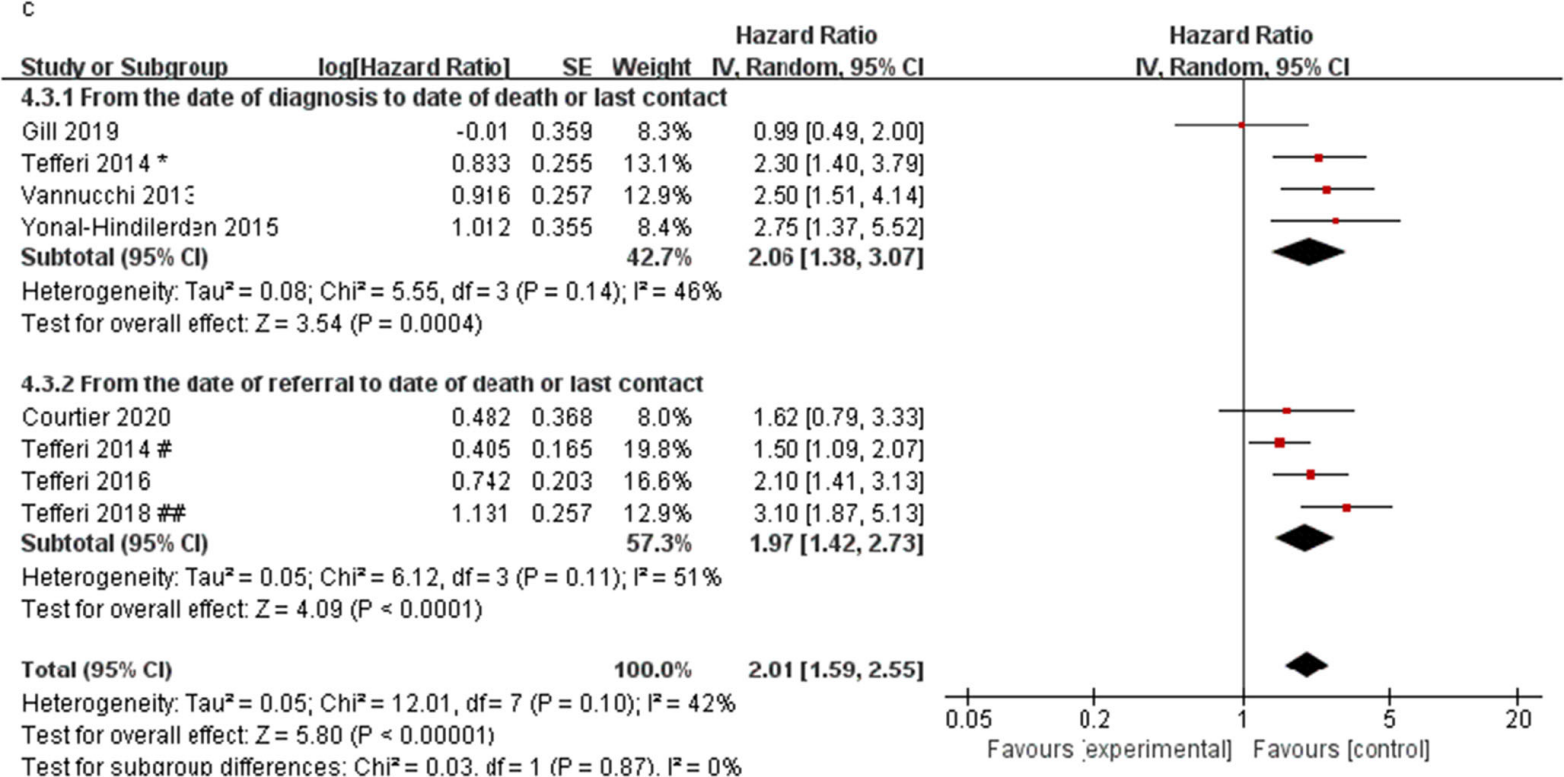
Forest plots of the pooled HRs and 95% CIs for subgroup analysis of OS in PMF patients with ASXL1 mutations stratified by different conditions in a random-effects model. **a** Stratified by sample size. **b** Stratified by region. **c** Stratified by OS definition methods. Number sign, one cohort of the study; single asterisk, another cohort of the study; double number sign, *British Journal of Haematology*; double asterisk, *Leukemia*

The results of the meta-analysis of the effect of ASXL1 mutations on OS in PMF patients showed that 13 studies have significant heterogeneity (*I*^2^ = 78%). We conducted subgroup analysis in terms of sample size, region, and OS definition methods, to check the heterogeneity and to determine whether the above factors will change the relationship between the ASXL1 mutations and OS. We observed a significant shorter OS in PMF patients with ASXL1 mutations than those with ASXL1 wild-type in both subgroups of sample size ≤ 200 (HR = 2.29, 95% CI: 1.55–3.37, *I*^2^ = 59%, *P* = 0.03) and sample size > 200 (HR = 2.30, 95% CI: 1.65–3.21, *I*^2^ = 86%, *P* < 0.00001) (Fig. 3a). According to the region of the studies, we found a significant association between ASXL1 mutations and OS in Europe (HR = 2.66, 95% CI: 2.05–3.47, *I*^2^ = 61%, *P* < 0.00001) and North America (HR = 2.06, 95% CI: 1.38–3.07, *I*^2^ = 66%, *P* = 0.0004), but not in Asia (HR = 2.21, 95% CI: 0.45–10.87, *I*^2^ = 87%, *P* = 0.33) (Fig. 3b). Furthermore, the definition method of OS did not alter the predictive value of ASXL1 mutations on the OS (from the date of diagnosis to date of death or last contact: HR = 2.06, 95% CI: 1.38–3.07, *I*^2^ = 46%, *P* = 0.0004; from the date of referral to date of death or last contact: HR = 1.97, 95% CI: 1.42–2.73, *I*^2^ = 51%, *P* < 0.0001) (Fig. 3c). The result of subgroup analysis suggested that the heterogeneity could not be explained by sample size, region, and the definition method of OS.

**Fig. 4:**
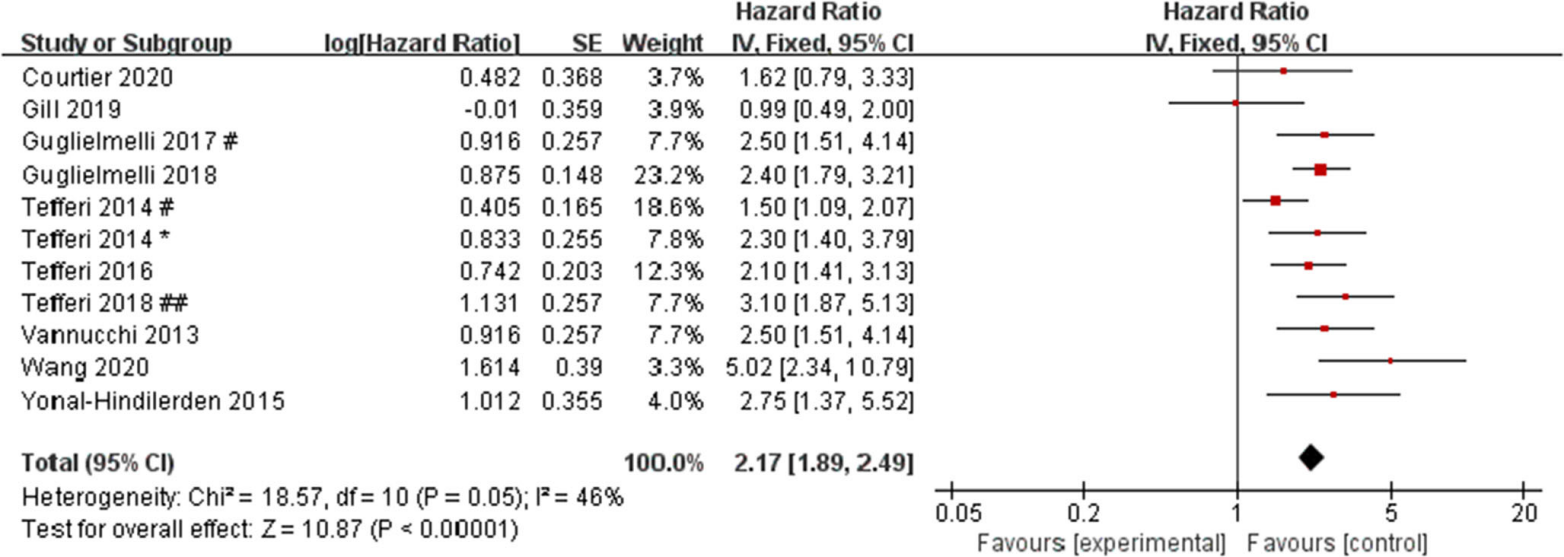
Forest plot of the pooled HRs and 95% CIs for OS in PMF patients with ASXL1 mutations (after removed the studies of Guglielmelli 2017 ^*^ and Tefferi 2018 ^##^) by a fixed-effects model. Number sign, one cohort of the study; single asterisk, another cohort of the study; double number sign, British *Journal of Haematology*; double asterisk, *Leukemia*

Examining the heterogeneity by removing one study at a time, it was found that when the data of overt-PMF patients in Guglielmelli (2017 ^*^) and Tefferi (2018 ^##^) were removed from the data queue, the remaining studies were significantly less heterogeneous than before (*I*^2^ = 46%), suggesting that these two studies were sources of heterogeneity. After further discussion of these two studies, we considered it because these two studies only included overt-PMF and DIPSS-plus low-risk and intermediate-1 risk PMF patients respectively. After removing these two studies, the pooled HR was 2.17 (95% CI: 1.89–2.49, *I*^2^ = 46%, *P* < 0.00001) (Fig. 4). The results still showed a significant association between ASXL1 mutations and OS, indicating that these two studies did not have a great impact on the results, so the random effect model was used to conduct the meta-analysis on 13 studies.

### Association between ASXL1 mutations and clinical features of PMF patients

**Fig. 5:**
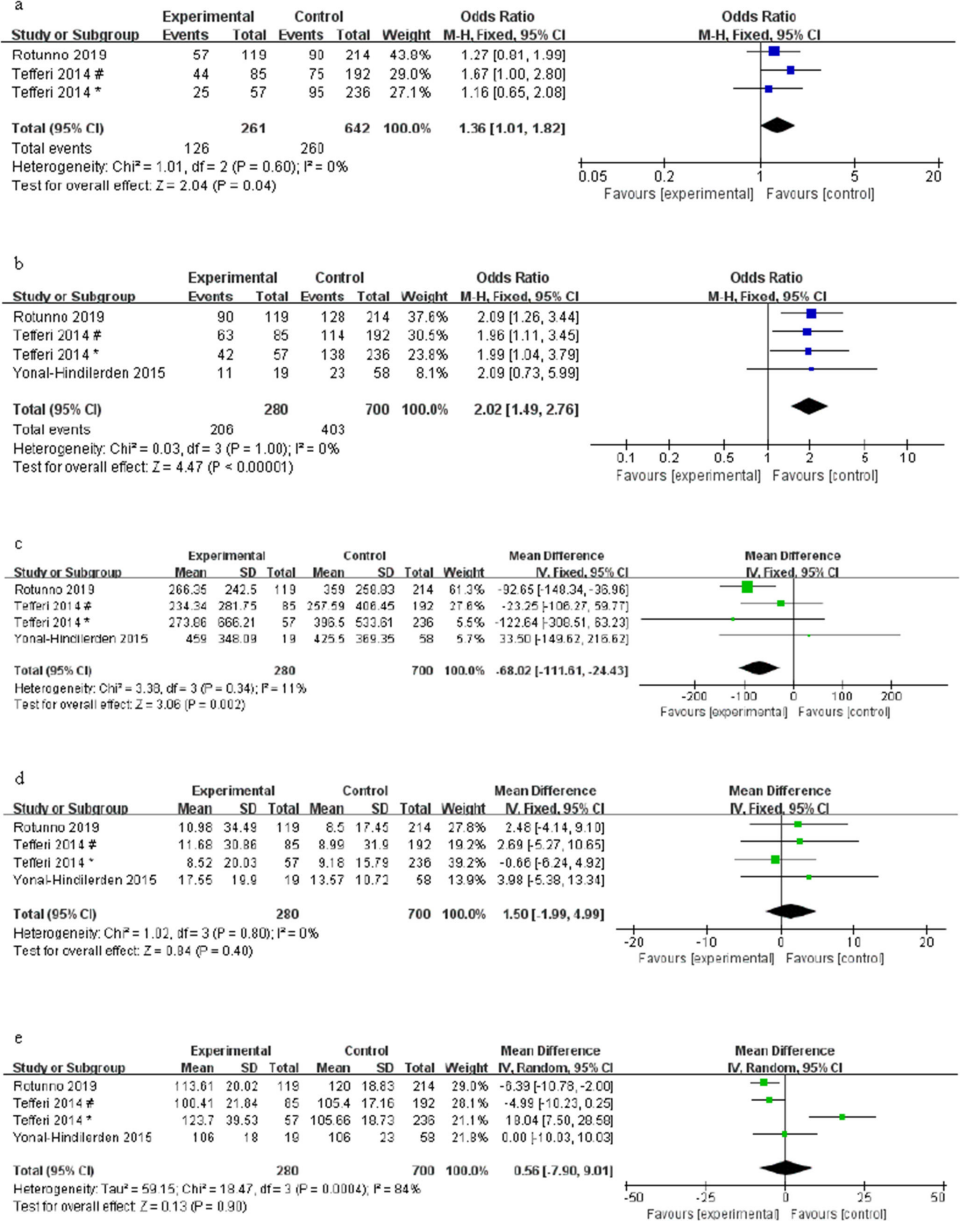

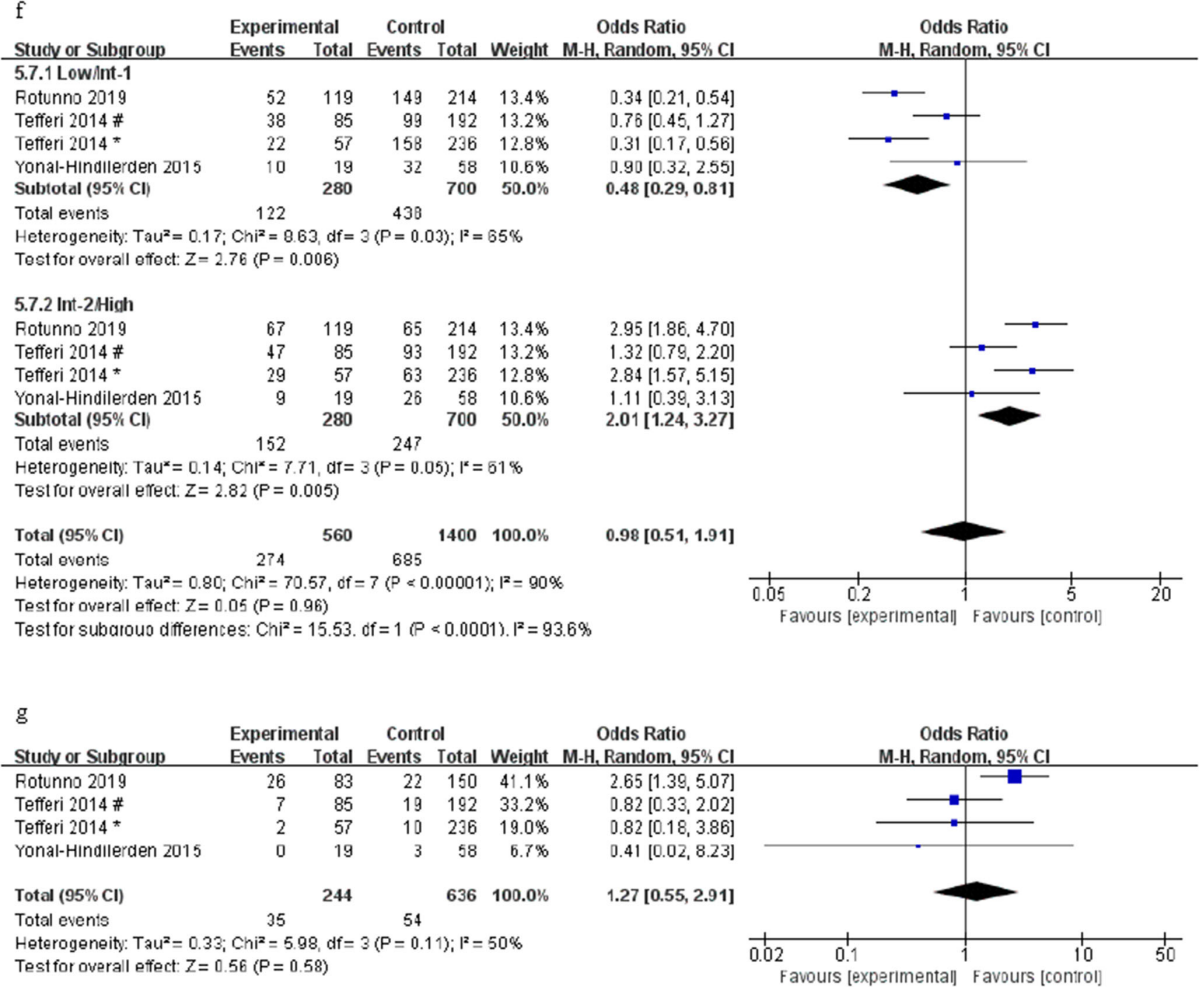
Forest plots for the association of different clinical features with ASXL1 mutations in patients with PMF by the fixed-effects model (**a**, **b**, **c**, **d**) and random-effects model (**e**, **f**, **g**). **a** Age > 65 years. **b** Gender. **c** PLT. d WBC. **e** HGB. **f** The risk of international prognostic score system. **g** Unfavorable karyotypes. Number sign, one cohort of the study; single asterisk, another cohort of the study

The meta-analysis was performed on the number of patients older than 65 years old and male patients with ASXL1 mutations and wild-type PMF, respectively, which found that age > 65 years and males are associated with ASXL1 mutations (OR = 1.36, 95% CI: 1.01–1.82, *I*^2^ = 0%, *P* = 0.04, Fig. 5a; OR = 2.02, 95% CI: 1.49–2.76, *I*^2^ = 0%, *P* < 0.00001, Fig. 5b). A total of four studies reported peripheral blood cell counts between ASXL1 mutations and ASXL1 wild-type PMF patients. Notably, our meta-analysis revealed that ASXL1 mutations were significantly related to a lower platelet count (MD = − 68.02, 95% CI: − 111.61 to − 24.43, *I*^2^ = 11%, *P* = 0.002) (Fig. 5c). No association was found between ASXL1 mutations and white blood cells or hemoglobin (MD = 1.50, 95% CI: − 1.99 to 4.99, *I*^2^ = 0%, *P* = 0.40, Fig. 5d; MD = 0.56, 95% CI: − 7.90 to 9.01, *I*^2^ = 84%, *P* = 0.90, Fig. 5e). In addition, we also found that ASXL1 mutations were significantly associated with a higher risk of the international prognostic score system (OR = 0.48, 95% CI: 0.29–0.81, *I*^2^ = 65%, *P* = 0.006; OR = 2.01, 95% CI: 1.24–3.27, *I*^2^ = 61%, *P* = 0.005) (Fig. 5f), but there was no statistical correlation between unfavorable karyotypes and ASXL1 mutations (OR = 1.27, 95% CI: 0.55–2.91, *I*^2^ = 50%, *P* = 0.58) (Fig. 5g).

### Sensitivity analysis and publication bias

**Fig. 6:**
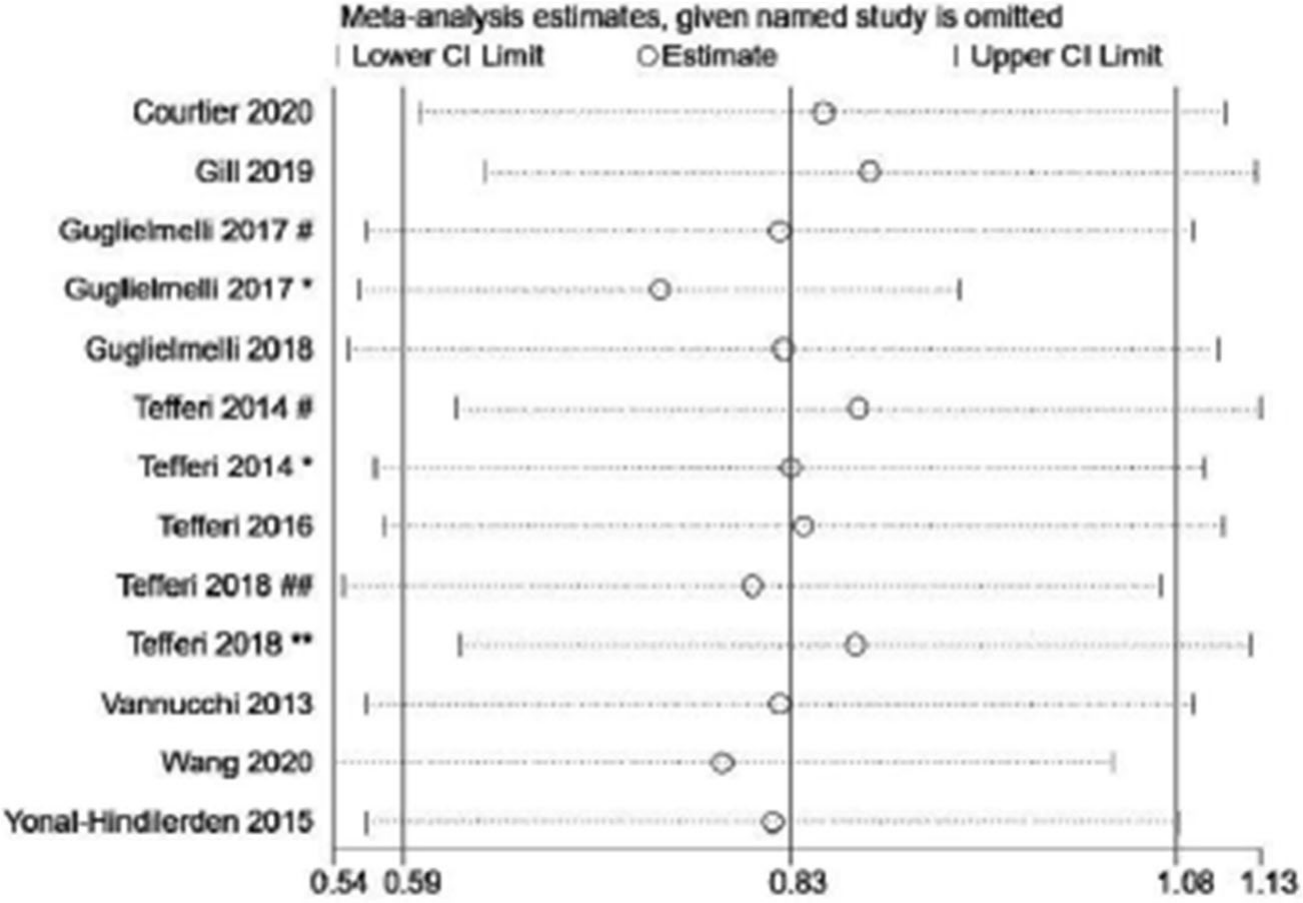
Sensitivity analysis for OS. Number sign, one cohort of the study; single asterisk, another cohort of the study; double number sign, *British Journal of Haematology*; double asterisk, *Leukemia*

**Fig. 7:**
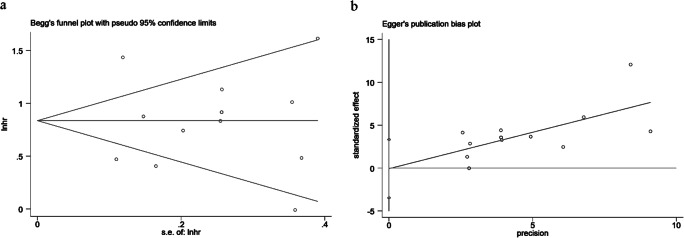
**a** Begg’s funnel plot. **b** Egger’s funnel plot for publication bias analysis of total OS

We conducted a sensitivity analysis of 13 studies describing the relationship between ASXL1 mutations and OS to validate the stability of the meta-analysis. As shown in Fig. 6, sensitivity analysis shows that no individual study had a predominant effect on the overall HR, indicating the results were stable and reliable. In addition, Begg’s and Egger’s tests were used to detect the publication bias, which indicated that there was no significant bias between studies (*P* = 0.272 of Begg’s test and *P* = 0.963 of Egger’s test) (Fig. 7).

## Discussion

PMF is a progressive malignant hematological disease with the worst prognosis in Ph-MPN, and its natural course is the transformation to acute myelocytic leukemia [1, 3]. A variety of clinical and biological characteristics such as advanced age, constitutional symptoms, blood cells and peripheral blood blasts, and chromosome karyotype, as momentous factors affecting the prognosis of PMF patients, have been included in the current prognostic score system [4–6]. The driver gene mutation status also has an important impact on the clinical manifestation, the rate of leukemia transformation, survival, and response to treatment of PMF patients [7–9, 37]. With the rapid development of sequencing technology, in addition to the driver genes, varieties of somatic gene mutations have been discovered in PMF patients [11–13], which may play a significant role in the pathogenesis and clinical prognosis of PMF patients. ASXL1 gene mutation is one of the representative mutations.

This meta-analysis covered 4501 PMF patients from different research centers, including 1393 PMF patients with ASXL1 mutations. The results revealed that ASXL1 mutations have a prominent adverse effect on the prognosis of PMF patients. The result is consistent with most previous research results. Tefferi A et al. [10] collected the information of 709 PMF patients who met the WHO diagnostic criteria, including driver gene mutation, ASXL1, or SRSF2 mutations, and unfavorable karyotype, to analyze their effects on OS and LFS in PMF patients, and univariate and multivariate analysis indicated that ASXL1/SRSF2 mutations predicted a poor prognosis. Some studies [16, 35] believe that ASXL1, EZH2, SRSF2, and IDH1/IDH2 are five “prognostically detrimental” mutated genes in PMF, and patients carrying any of these mutated genes belong to the high-molecular risk category (HMR); such patients have shortened OS and increased prevalence of AML. Moreover, we found that ASXL1 mutations were more likely to occur in patients who were older than 65 years old, males, with a lower platelet count, or intermediate-2 risk and high-risk of the international prognostic score system, in accord with the results of Cervantes F et al. [4] and Gangat N et al. [6], which also explain to a certain extent why the ASXL1 mutations have an adverse effect on the prognosis of PMF patients. Due to the lack of data, we were unable to evaluate all clinical features. The results of subgroup analysis manifested that the Asian studies did not show a correlation between ASXL1 mutations and OS, and the remaining results were in keeping with the overall results. However, studies conducted in Asian populations found that ASXL1 mutations are an independent unfavorable prognostic factor for OS in other myeloid malignancy [38]. Since there were only two studies from Asia and the sample size was small, it is necessary to perform a retrospective or prospective cohort study with a large number of patients.

The heterogeneity of overall OS meta-analysis results was high, which could not be explained by sample size, region, and OS definition methods. Examining the heterogeneity by removing one study at a time, it was found that when the studies of Guglielmelli (2017 ^*^) and Tefferi (2018 ^##^) were excluded, the heterogeneity of the remaining studies was lower than before, suggesting that these two studies are the sources of heterogeneity. After further discussion, we considered that the heterogeneity was related to the inclusion of overt-PMF and DIPSS-Plus low-risk and intermediate-1 risk PMF patients respectively in these two studies. After the removal of the two studies, it was still indicated that ASXL1 mutations had a significant correlation with OS, and the sensitivity analysis did not show any studies had a significant impact on the results. Therefore, the conclusion of this meta-analysis is relatively reliable.

ASXL1 encodes a nuclear protein with a length of 1541 amino acids, whose main function is to regulate epigenetics and transcription [39]. However, the exact mechanism of ASXL1 mutation in the pathogenesis of PMF is still unclear, but there are still researches devoted to exploring. ASXL1 mutations lead to the loss of trimethylation of histone H3 lysine 27 (H3K27) mediated by polycomb repressive complex 2 (PRC2), thereby promoting myeloid transformation [40]. Balasubramani et al. [41] reported that the mutant protein of C-terminally truncated ASXL1 can enhance the deubiquitinating enzyme activity of BAP1 and affect the differentiation of myeloid hematopoietic cells. ASXL1 mutations alter the epigenome of hematopoietic stem cells (HSC) and increase the susceptibility to leukemia transformation [42]. In hematological malignancies, C-terminally truncated ASXL1 mutant protein significantly weakens the transcriptional regulation of BAP1-ASXL1-FOXK1 / K2 complex, downregulates the expression of multiple tumor suppressor genes, and then regulates glucose metabolism, hypoxia perception, JAK-STAT, and other tumor-related signaling pathways, promoting proliferation and self-renewal of leukemia cells [43]. Therefore, ASXL1 mutations may play an important role in the pathogenesis and transformation of PMF. ASXL1 mutations include three types: frameshift mutations, nonsense mutations, and missense mutations. Due to the lack of data, this study failed to specifically analyze the relationship between ASXL1 mutation types and the prognosis of PMF patients. Studies have evaluated the impact of the mutation type of ASXL1 mutation in PMF on the prognosis, and the results indicate that compared with the unmutated state of ASXL1, the three major mutants (frameshift, nonsense, and missense) have significant disadvantageous effects on prognosis [19], which is in line with the results of our study. In the future, it is still necessary to design more reasonable experiments to explore the impact of ASXL1 mutations on the pathogenesis of PMF. In clinical, attention should also be paid to the relationship between ASXL1 and its different mutation types and clinical characteristics as well as prognosis, so as to improve the accuracy of risk stratification and prognosis assessment.

Despite our efforts to perfect this meta-analysis, it still has its own limitations. First of all, the articles we included are all retrospective, observational studies, rather than prospective randomized controlled trials, and the selection criteria are difficult to grasp; the homogeneity of the researches is hard to guarantee; secondly, although we conducted a comprehensive search of the literature in the database, Begg and Egger tests suggested there was no obvious publication bias, but we only covered studies published in English, which could not completely avoid publication bias; thirdly, the heterogeneity of OS meta-analysis results was a little large in both the overall and subgroups, which may have something to do with the different clinical characteristics of each study. Furthermore, many HRs were the result of multivariate analysis in the included studies, but the confounding factors were different in each study, which may also be the source of heterogeneity.

In conclusion, this meta-analysis revealed that ASXL1 mutation had a significant adverse effect on the prognosis of PMF patients, and PMF patients with ASXL1 mutations had a shorter OS and a greater possibility of transformation to leukemia. In addition, ASXL1 mutations were associated with the age of > 65 years, males, a lower platelet count, and a higher risk of international prognostic score. Based on the current research, ASXL1 mutation is expected to become a new molecular marker for the risk stratification and prognosis assessment of PMF patients. But before that, a prospective cohort study covering a large sample size is needed to provide a more reliable basis for the relationship between the ASXL1 mutations and the prognosis of PMF patients. We believe that with the in-depth study of genetic variation and the development of next-generation sequencing technology, our understanding of the pathogenesis and risk factors of hematological malignancies will be more profound.
